# Finite element analysis of changes in tensile strain and deformation by airbag impact in eyes of various axial lengths

**DOI:** 10.1007/s10792-022-02609-7

**Published:** 2022-12-19

**Authors:** Ayaka Kobayashi, Ryosuke Izaki, Hideaki Fujita, Kazuhiro Harada, Hiroaki Ozaki, Kazuaki Kadonosono, Eiichi Uchio

**Affiliations:** 1 Department of Ophthalmology, Fukuoka University School of Medicine, 7-45-1 Nanakuma, Jonan, Fukuoka, 814-0180 Japan; 2 Department of Ophthalmology, Yokohama City University Medical Center, 4-57 Urafune-cho, Minami, Yokohama, 232-0024 Japan

**Keywords:** Airbag, Ocular trauma, Computer simulation, Cornea, Deformation, Finite element analysis

## Abstract

**Purpose:**

Airbags have substantially reduced mortality and morbidity, while ocular injuries caused by airbags have been reported. We applied a three-dimensional finite element analysis (FEA) model we have established for evaluation of the deformation of an intact eyeball of various axial lengths induced by an airbag impact at various impact velocities.

**Methods:**

A model human eye we have created was used in simulations with an FEA program, PAM-GENERIS™ (Nihon ESI, Tokyo, Japan). The airbag was set to impact eyes with various axial lengths of 21.85 mm (hyperopia), 23.85 mm (emmetropia) and 25.85 mm (myopia), at initial velocities of 30, 40, 50 and 60 m/s. Changes in the shape of the eye and the strain induced were calculated. Deformation of the eye in a cross-sectional view was displayed sequentially in slow motion.

**Results:**

We found that considerable damage, such as corneal or scleral lacerations, was observed especially at higher impact velocities, such as 50 or 60 m/s, in eyes with any axial length. Deformation was most evident in the anterior segment. The decrease rate of axial length was greatest in the hyperopic eye, followed by the myopic eye, and the emmetropic eye.

**Conclusions:**

It was shown that hyperopic eyes are most susceptible to deformation by an airbag impact in this simulation. The considerable deformation by an airbag impact on the eye during a traffic accident shown in this study might indicate the necessity of ocular protection to avoid permanent eye damage.

## Introduction

Airbags have saved thousands of lives since their introduction in the early 1980s. Airbags protect passengers from a crash by providing a padded device that allows the impacting and impacted surface to deform and thereby extends the duration of the impact and reduces its severity [1]. Although airbags have substantially reduced mortality and morbidity, those who survive may suffer from various fatal and nonfatal injuries of the head, eyes, neck, chest, or arms [2, 3]. An airbag impact can result in severe ocular damage, significant loss of vision, and, in extreme cases, complete loss of the eye [4]. Ocular injuries caused by airbags have been widely reported in the literature [5–16], and most injuries require immediate emergency surgery and numerous follow-up surgeries.

However, only a few studies have evaluated the clinicopathological mechanism of airbag ocular injuries, especially in a simulation model [17]. We have previously developed a simulation model resembling a human eye based on the information obtained from cadaver eyes and applied 3-dimensional finite element analysis (FEA) to determine the physical and mechanical conditions of impacting foreign bodies that cause an intraocular foreign body [18]. This model human eye was also used in our studies on airbag impact in various situations [19–22].

Therefore, we planned to study the kinetic phenomenon of an airbag impact on a human eye using a FEA method, which was also applied in several of our previous studies on other types of injury than airbag injury [23, 24]. If we were able to reproduce the kinetic phenomena including intraocular deformation caused by an airbag impact sequentially, this would increase understanding of the pathophysiological mechanism of blunt ocular trauma by an airbag.

In this study, we extended the simulation model to further determine the physical and mechanical response of intact eyes with different axial lengths to an impacting airbag deploying at various velocities, especially surveying the mechanical threshold in a hyperopic or myopic eye.

## Materials and methods

A model human eye was created and used in simulations with a computer using an FEA program, PAM-GENERIS™ (Nihon ESI, Tokyo, Japan), described elsewhere [18]. The elastic properties and the meshing principles of the model human eye were similar to those in previous reports [18, 20]. Poisson ratio of the cornea of 0.420 and the sclera of 0.470 were used to determine the standard stress strain curves for cornea and sclera [25, 26]. A vitreous model as a solid mass with a hydrostatic pressure of 20 mmHg (2.7 kPa) was also assigned [18]. The anterior chamber was set at a depth of 5.1 mm, the vitreous length was assumed to be 18.6 mm, and the posterior curvature of the retina was assumed to be 12.0 mm in the emmetropic eye model [18].

**Fig. 1:**
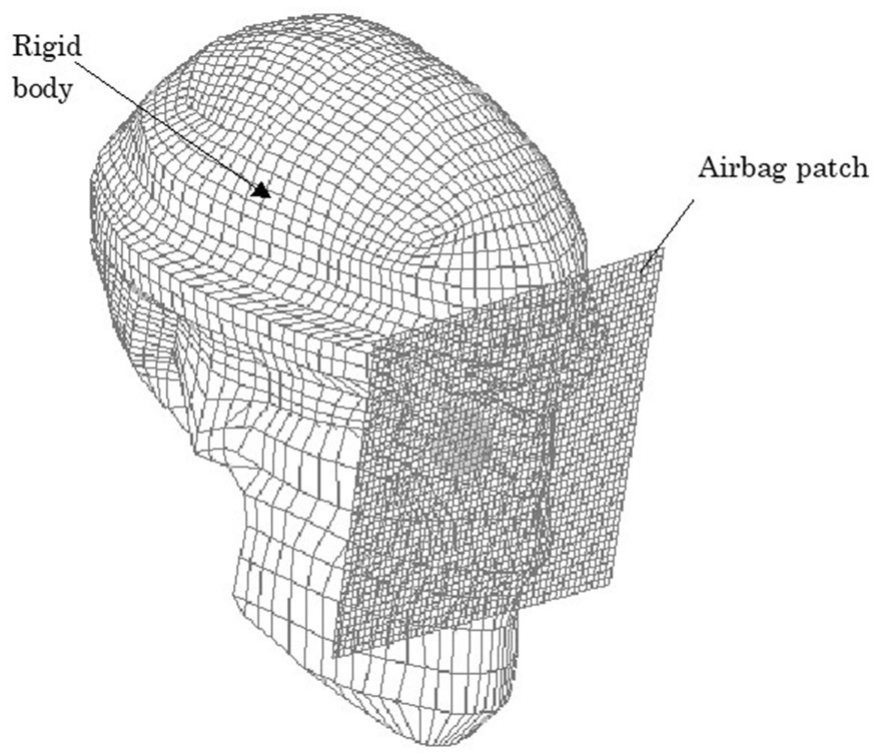
Biomechanical model of the head. Parts other than the eye were assumed to be rigid elements, and the impacting object was placed adjacent to the eyeball to reduce the computing time of the airbag impact simulation

The first step involved modifying the Hybrid III model [27] by replacing the head of the dummy with a biomechanical model of the head in which the eyeball model was inserted, as shown in Fig. 1, assuming that everything excluding the eye was a solid element, to reduce the computing time [18]. The industry average for the deploying velocity of an airbag is 64.5 m/s [28]. Airbag deployment velocities have been calculated to range from 100 to 300 km/h (approximately 28 to 83 m/s) [29]. Although the deployment velocity of an airbag can be calculated or is shown in the specification of each car model, impact velocities of the airbag to the eye are unclear. In standard car crash tests (a car hitting a wall at 50 km/h), airbag inflation is initiated 15 ms after the crash, and the airbag finishes expanding and briefly maintains a constant volume before drawing off of the gas from exhaust holes [8]. According to these data, for comparison of various airbag impact velocities and to determine the threshold velocity causing corneal rupture, impact velocities of the airbag patch on the face were 30, 40, 50 and 60 m/s, which are values in the range of suspected airbag deployment velocities as shown above. The reference point for globe rupture was then calculated to be at a strain of 18.0% and stress of 9.45 MPa for the cornea, and at a strain of 6.8% and stress of 9.49 MPa for the sclera, which exceeded the tensile tolerance based on element deletion method [18]. Different axial lengths each represent; a normal eye with a normal axial length of 23.85 mm, a hyperopic eye with a shorter axial length of 21.85 mm, and a myopic eye with a longer axial length of 25.85 mm. Eyes with different axial lengths were created by setting the mass density of the cornea and sclera as constants and the element types including the three layers of the model eye: outer, middle, and inner, as variables for meshing principles [18]. In each model, ocular elements were shortened or elongated according to the proportion against the normal eye (emmetropia) for optic axis direction, but diameters in the frontal view and total volume of the cornea and sclera were assumed to be constant.

**Fig. 2:**
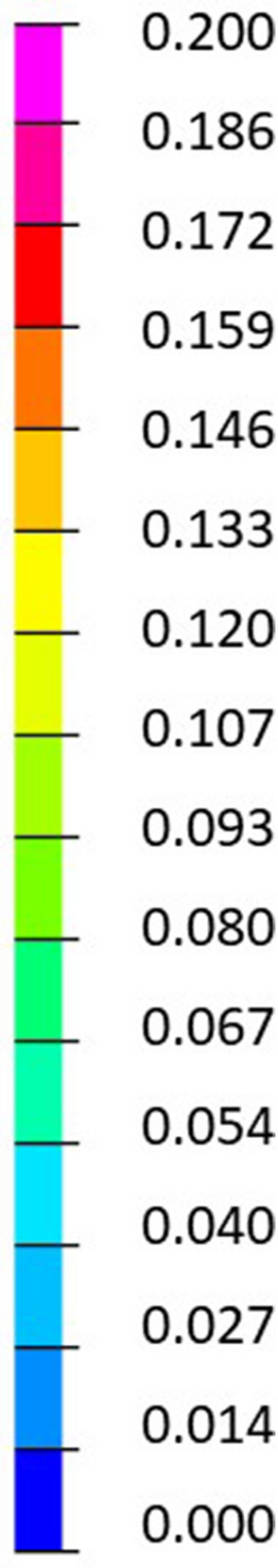
Deformation scale in simulation. Color mapping scale of deformation of eye showing strain induced; warmer color of red represents greater deformation

Changes in the shape of the eye and the strain induced were calculated using Virtual Performance Solver (VPS) (Nihon ESI) and evaluated by color mapping (Fig. 2). Deformation of the eye in a cross-sectional view was displayed sequentially in milliseconds in slow motion.

## Results

**Fig. 3:**
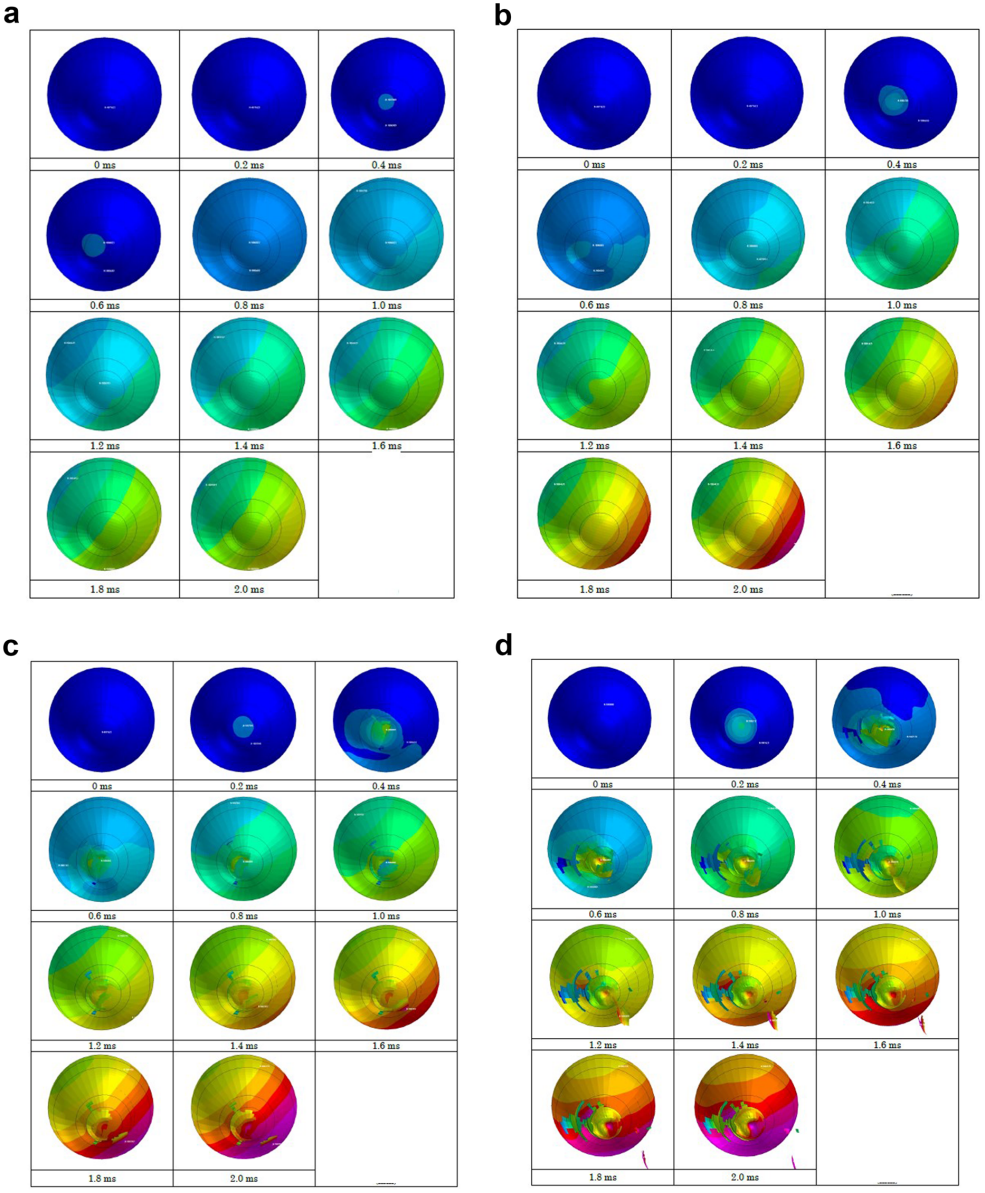
Sequential deformity of normal axial length model eye upon airbag impact at four different velocities, shown at 0.2-ms intervals. Cases of impact velocity of 30 (**A**), 40 (**B**) 50 (**C**) and 60 m/s (**D**) in normal length model eye are shown. Strain strength change is displayed in color as presented in the color bar scale (Fig. 2).

**Fig. 4:**
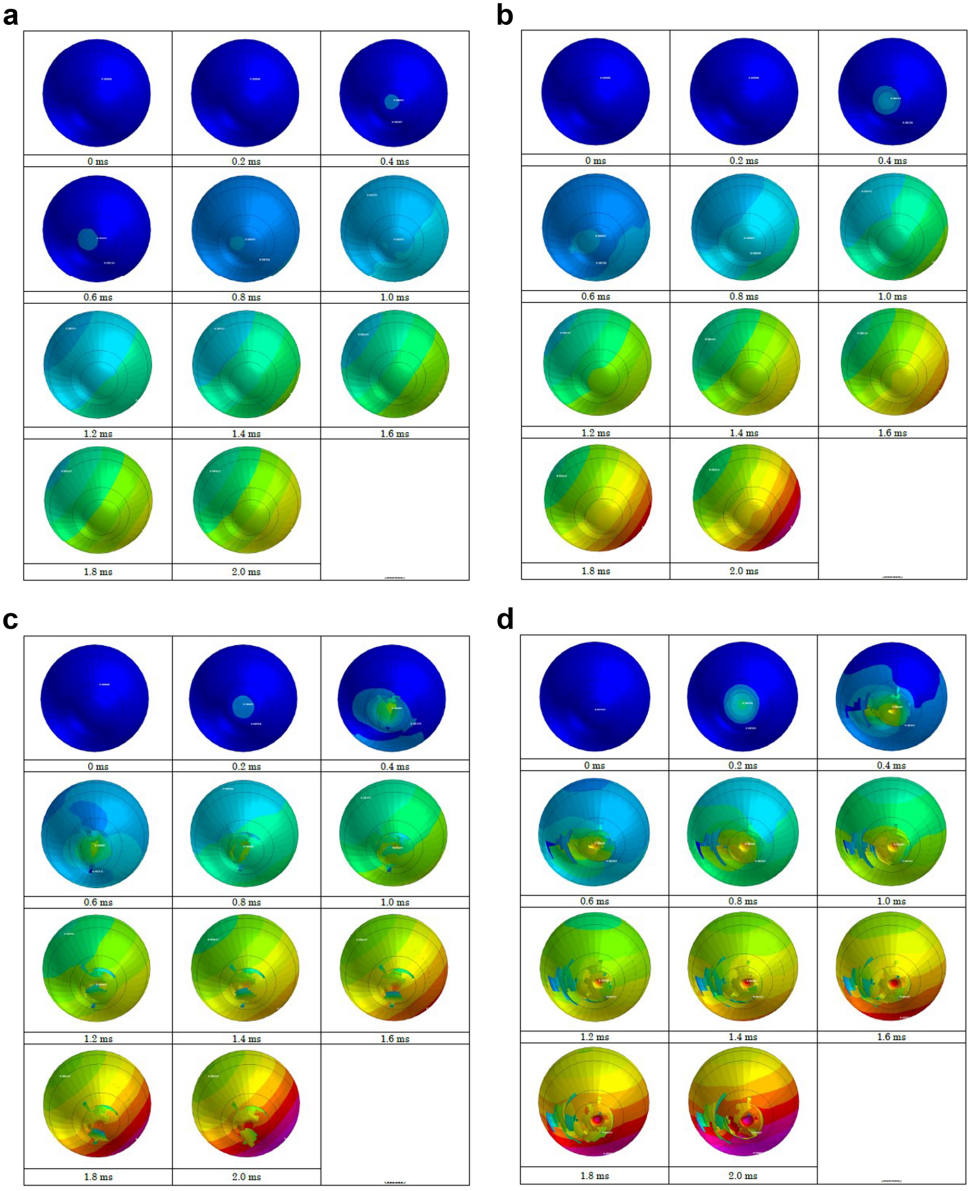
Sequential deformity of long axial length (myopic) model eye upon airbag impact at four different velocities, shown at 0.2-ms intervals. Cases of impact velocity of 30 (**A**), 40 (**B**) 50 (**C**) and 60 m/s (**D**) in long axial length model eye are shown. Strain strength change is displayed in color as presented in the color bar scale (Fig. 2)

**Fig. 5:**
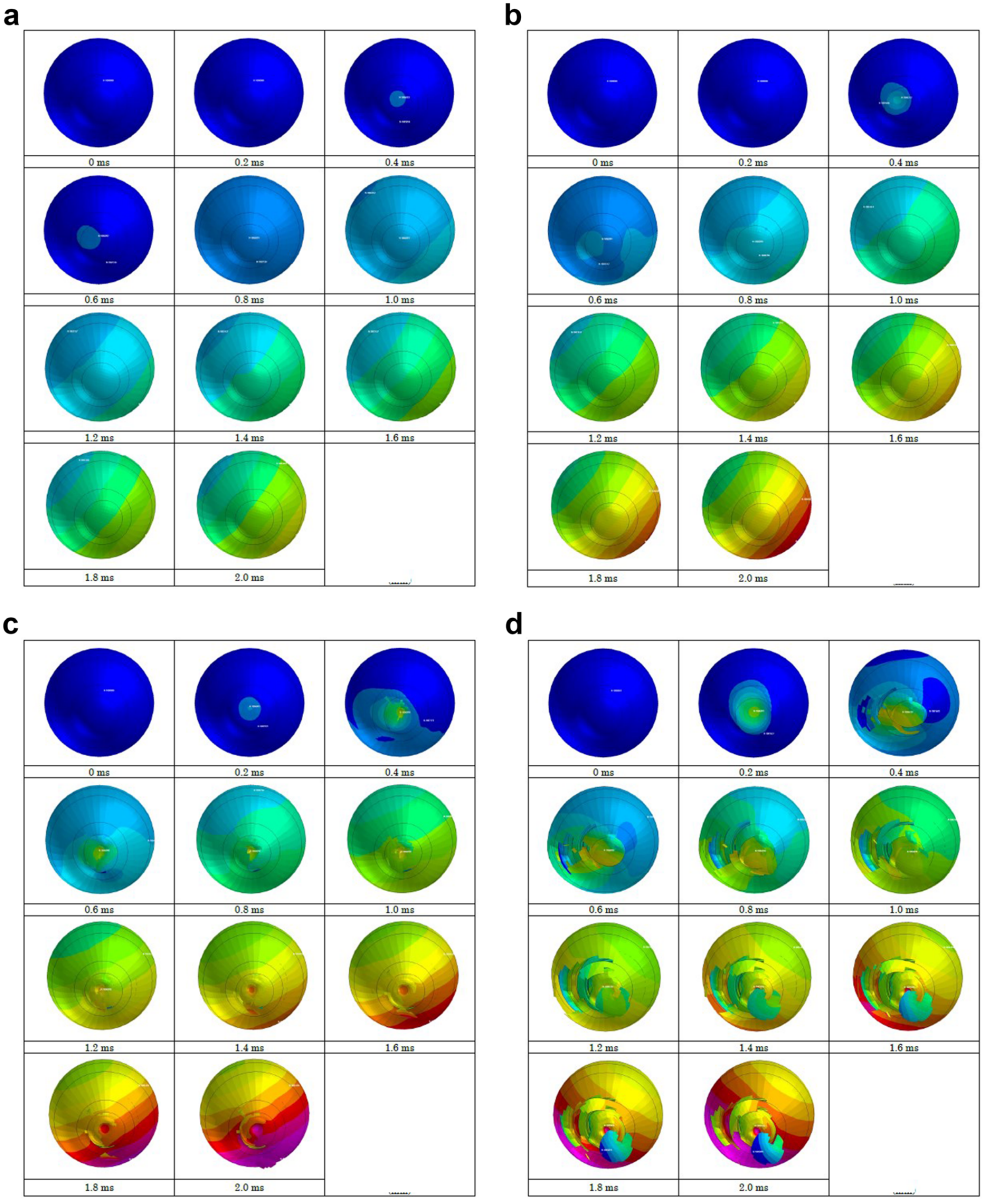
Sequential deformity of short axial length (hyperopic) model eye upon airbag impact at four different velocities, shown at 0.2-ms intervals. Cases of impact velocity of 30 (**A**), 40 (**B**) 50 (**C**) and 60 m/s (**D**) in short axial length model eye are shown. Strain strength change is displayed in color as presented in the color bar scale (Fig. 2)

Strain strength response of the ocular surface, cornea and sclera to an airbag impact was evaluated in eyes of normal axial length, shorter axial length and longer axial length. In the case of an impact velocity of 30 m/s, corneal strain did not reach its threshold (18.0%) at the end of simulation (2.0 ms) after the impact for all axial lengths (Figs. 3-A, 4-A, 5-A). When the airbag impacted at 40 m/s, the cornea might have reached its strain strength threshold transiently after 1.8 ms, and the sclera also reached its threshold similarly at 1.6 ms after airbag impact in all axial length models (Figs. 3-B, 4-B, 5-B). In the impact velocity of 50 m/s simulation, scleral laceration occurred at 1.0 ms after the impact in all length models in simulation; in contrast, while corneal deformity reached its threshold at 1.8 ms in the longer axial length eye, corneal laceration was observed at 1.6 ms in the normal length eye and at 1.8 ms in the shorter axial length eye (Figs. 3-C, 4-C, 5-C). The critically damaged area of the ocular surface was largest in the shorter length eye, followed by the normal length eye, and the longer length eye, at the end of the 50 m/s impact velocity simulations. In cases of 60 m/s impact velocity, scleral strain reached its threshold at 0.8 ms after the impact in all three axial length models, and the proportion of the suspected scleral laceration area was largest in the shorter length eye considering the area below the threshold. Corneal laceration began at 1.4 ms after the impact in all axial length eyes, and seamless area damage adjacent to the sclera was most apparent in the normal length eye in the simulation. In the shorter length eye model, an irregular image in the front of the cornea was recorded that might be due to tissue damage resembling corneal perforation (Figs. 3-D, 4-D, 5-D).

**Fig. 6:**
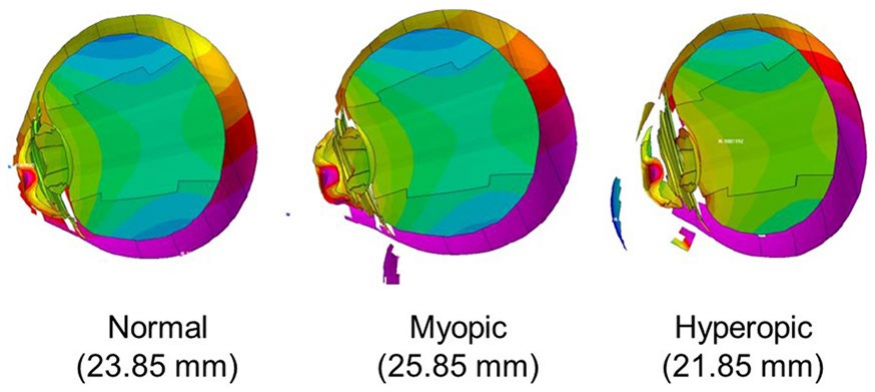
Sagittal view of model eye at 2.0 ms after airbag impact at velocity of 60 m/s for eyes with three axial lengths

Sagittal views of the model eye at 2.0 ms after an airbag impact at a velocity of 60 m/s in the three axial length eyes are shown in Fig. 6. Deformity of the intraocular segment, such as the anterior chamber, lens and vitreous body, could be visualized in this view. Deformity of vitreous was less than that of the lens and anterior chamber, especially in the hyperopic eye. The degree of retinal and posterior scleral deformity was most apparent in the hyperopic eye, followed by myopic and normal eyes (Fig. 6). The decrease rate of axial length (percentage of shortened axial length due to airbag impact in normal axial length) in each axial length model is shown in Table 1. The decrease rate was largest in the hyperopic eye, followed by the myopic eye, and the emmetropic eye. These results indicate that hyperopic eyes are most susceptible to deformation by an airbag impact compared with other axial length eye models in this simulation.

**Table 1:** Percent decrease of axial length at 2.0 ms after airbag impact

Impact velocity (m/s)	30	40	50	60
*Axial length status*				
Emmetropia (23.85 mm)	6.7%	11.1%	11.1%	13.4%
Hyperopia (21.85 mm)	12.0%	16.7%	19.0%	21.4%
Myopia (25.85 mm)	10.2%	12.3%	14.2%	16.3%

## Discussion

We have previously applied this simulation model to ocular injury by an airbag impact in several studies [20–22]. In these reports, a surgical procedure was added in the simulation eye model, such as radial keratotomy [20], photorefractive keratectomy [21] or transscleral fixation of a posterior chamber intraocular lens [22]. In contrast, this report examined a series of airbag-induced injury in our simulation studies performed on an intact eye, which comprises most cases suffering from airbag deployment in clinical situations. In this study, we reported that considerable damage, such as corneal or scleral laceration, was observed especially at higher impact velocities, such as 50 or 60 m/s, at any axial length. This seems reasonable because greater physical energy is derived from a higher airbag impact velocity. It has been reported that airbag-related eye injuries occurred very rarely in car accidents in cases where the occupant survived and the restraint system was appropriately used [14], and airbags had no significant effect to increase eye injuries in studies on motor vehicle collisions [30]. Hwang et al. recommended that when using airbags, seat belts should be used together, because seat belts were effective to decrease eye injuries in motor vehicle collisions [30]. From these studies, serious ocular injuries induced by airbag deployment in car accidents are expected to be limited to cases such as improper use of a restraint system or passengers with short stature.

A limited number of studies have evaluated the relationship between axial length and severity of blunt ocular trauma [31–33]. Rau et al. reported in an experimental study using human cadaveric eyes that the force at corneal rupture was not associated with axial length [32]. However, Hashemi et al. reported that axial length was significantly longer in cases with a history of trauma in a cross-sectional study of a clinical database of ocular trauma cases [31]. Even considering the difference in design of these studies, their results were contradictory. Therefore, we have carried out several FEAs on blunt ocular trauma impact by an airbag or airsoft gun in eyes of various axial lengths using a simulation eye model we have established [22, 24]. While hyperopic eyes are most susceptible to deformation by an airsoft gun impact compared with other axial length eye models [24], eyes with long axial length (myopic) experienced greater deformity upon airbag impact [22]. The reason for this discordance is unclear, but it might be due to difference in outcome measures between these studies, because the latter study focused on the upper limit of the impact force exceeding the tensile force of 10–0 polypropylene that caused breakage of sutures [22]; in contrast, the former report mainly evaluated strain strength threshold of the cornea and sclera [24].

As indicated above, the results of the present study are consistent with our previous study of an FEA model to evaluate airsoft gun impact on an eye and the deformation rate of eyes of various axial lengths at various velocities [24]. It is also interesting that deformity of posterior segments including the vitreous body and retina was less than that of the anterior chamber, especially in the hyperopic eye, in sagittal images (Fig. 6); however, the degree of retinal or posterior scleral deformity was most apparent in the hyperopic eye (Fig. 6). Our previous report of airsoft gun impact in which deformation was most evident in the anterior segment, while deformation of the posterior segment was less, also supports the results of this study. The degree of tissue strain or area of deformation was greatest in the shorter axial eye followed by the normal eye and the longer axial eye compared at the same airbag impact velocity in the simulation in this study; however, the difference seems not so evident. The impact velocities set in this simulation study were within those of current vehicles and were chosen linearly; thus, the results obtained did not show apparent differences. In clinical situations, other factors, such as head movement, might add further irregular physical impact on the eyeball; however, these were not included in the variables in this simulation. These could be the reasons for the lack of an evident tendency of ocular deformation in this study. Considering the rare occurrence of airbag-related eye injuries, if the restraint system was used appropriately [14], our results seem consistent with the rarity of serious ocular blunt injury caused by airbag impact. However, the present result that the cornea and sclera might suffer considerable strain that might result in laceration of the eyeball at higher impact velocities (50 or 60 m/s) emphasized the necessity of wearing a seatbelt or eye protection, such as glasses, to prevent serious ocular injury especially in high-risk populations, such as short-stature persons or small children in the passenger seat. If future innovations, such as automatic notification warning to raise seat height or automatic seat-height correction system to prevent direct impact by deploying airbag to eyeballs of these high-risk passengers, were introduced in automobile safety mechanism, serious airbag-induced ocular injury would be expected to decrease.

As for decrease rate of axial length, the present result was not inconsistent with our previous study of airsoft gun impact simulation [24]. The highest decrease rate was observed in the hyperopic eye in both studies; however, it was followed by the myopic eye in this study (Table 1), while the emmetropic eye was the second highest in the airsoft gun simulation study [24]. It is difficult to determine the exact reason; however, the larger area of compression and higher energy with airbags caused a more considerable effect on the myopic eye, in which ocular wall thickness became thinner according to the extension of the volume of the eyeball, compared with the normal length eye with a thicker ocular wall.

Despite our careful calculation in the simulation model, there are still limitations to our study. First, in order to reduce the time for computer calculation, the impacting object was placed almost adjacent to the surface of the eyeball for impact simulation. Therefore, further study on the distance of the deploying airbag from the driver in regard to the change in velocity would be necessary to determine the impact that is likely to occur in reality. Secondly, the vitreous model was a solid mass with physiological intraocular pressure of 20 mmHg [18]. Factors associated with aging such as vitreous viscosity and other physical characteristics were not considered as variables in this simulation. However, we believe that our goal to assess the mechanical properties of eyes with different axial lengths impacted by an airbag was achieved by demonstrating that corneoscleral laceration might occur at higher impact velocity in eyes with shorter axial length, being more susceptible to impact injuries.
